# Knowledge, Attitude, and Practice of the Head Impulse, Nystagmus, and Test of Skew (HINTS) Examination Among Emergency Physicians in Saudi Arabia: A Cross-Sectional Study

**DOI:** 10.7759/cureus.80366

**Published:** 2025-03-10

**Authors:** Essa S Albalawi, Ahmed Esmael Abdelhady, Ibrahim Hadi Alsroujy, Jumanah Yaser Nassar, Majduldeen Azzo

**Affiliations:** 1 Emergency Department, International Medical Center, Jeddah, SAU; 2 Emergency Department, East Jeddah Hospital, Jeddah, SAU; 3 College of Medicine, Ibn Sina University, Jeddah, SAU; 4 Emergency Department, Eastern Health, Victoria, AUS

**Keywords:** attitude, emergency physicians, hints, knowledge, practice

## Abstract

Objectives

We aimed to assess the knowledge, attitude, and practice levels of the head impulse, nystagmus, and test of skew (HINTS) examination by the Saudi Commission for Health Specialties registered emergency physicians and board residents in Saudi Arabia.

Materials and methods

This cross-sectional survey study enrolled 101 registered emergency physicians and board residents in Saudi Arabia. Participants completed a self-administered online survey evaluating their understanding, attitudes, and application of the HINTS examination, a tool to differentiate central from peripheral vertigo.

Results

A high proportion of participants (68/101, 67.3%) demonstrated a satisfactory level of knowledge, exhibited a favorable attitude (59/101, 58.4%), and engaged in good practice (82/101, 81.2%). However, the majority of physicians expressed disagreement with the use of the HINTS examination to differentiate between central and peripheral vertigo. The HINTS examination is underutilized by most emergency physicians (56/101, 55.4%) and during consultations (47/101, 46.5%). There was a statistically significant association between position, certifications, and knowledge (p=0.017, 0.028), while certification was the sole statistically significant predictor of attitude (p=0.004).

Conclusions

Physicians with less than five years of experience, particularly those in training or holding the Saudi Board of Emergency Medicine certification, demonstrated stronger knowledge, more favorable attitudes, and greater utilization of the HINTS examination. Identified gaps in the exam's perceived sensitivity, appropriate use, and physicians’ confidence highlight the need for additional educational interventions to promote effective implementation in emergency settings.

## Introduction

The head impulse, nystagmus, and test of skew (HINTS) examination has been demonstrated to have 100% specificity and 96% sensitivity for stroke in specialized units when performed by neurologists and neuro-otologists with the necessary training [1]. It is a bedside diagnostic test to rule out a central source of vertigo in patients presenting with acute vestibular syndrome (AVS) [2]. It can identify stroke as the underlying cause of AVS within the initial 48 hours following the manifestation of symptoms [3]. Moreover, it is most useful in patients with continuous, ongoing vertigo [4]. The HINTS examination assists clinicians in determining the necessity of neuroimaging with a higher degree of sensitivity than MRI. Consequently, it reduces wait times and healthcare expenditures in emergency departments by decreasing the frequency of unnecessary imaging procedures [2]. However, research indicates that emergency medicine (EM) doctors rarely utilize the HINTS examination, and when they do, it is typically applied, interpreted, and recorded in an improper manner [2,5].

Emergency physicians hold disparate opinions about the HINTS examination. Some emergency physicians believe it is essential for them to gain familiarity with, practice, and become proficient in the HINTS examination, as it is crucial in determining posterior circulation stroke in patients with AVS [6]. However, due to a lack of necessity or validation of the test among emergency physicians, some doctors may choose not to use the HINTS examination [7].

EM is a relatively new medical specialty in Saudi Arabia, receiving only formal recognition in 2001. The Saudi Board of EM was established in 2005 to create, implement, and evaluate a unified curriculum for EM residents. Since then, EM in Saudi Arabia has expanded and changed [8].

To our knowledge, no Saudi survey has been conducted among emergency physicians to determine their awareness and use of HINTS examination. Therefore, the current study aimed to assess the knowledge, attitude, and practice level regarding the HINTS examination among the Saudi Commission for Health Specialties (SCFHS)-registered emergency physicians and board residents in Saudi Arabia.

## Materials and methods

Ethical considerations

This study was approved by the Ethics Committee of the Ministry of Health, Saudi Arabia (IRB no: 24-33 M, date: 21/3/2024). Prior to their participation, each subject provided a written consent after being informed of the study's goals and methods. We ensured that participants’ confidentiality was protected.

Design, setting, and duration

This cross-sectional study was conducted at central and western Saudi Arabia hospitals from 22/3/2024 to 18/5/2024.

Participants

This study included all EM residents enrolled in the Saudi Board training program. Additionally, senior emergency physicians classified by the SCFHS, including registrars, senior registrars, and consultants, were also eligible for participation. Physicians who were not formally registered as residents under the SCFHS EM board training program were excluded at the time of the study. This included non-board-certified emergency physicians, general practitioners, physicians holding the title of "resident" at their institution but not officially enrolled in the SCFHS training program, and physicians working in institutions not designated as training centers by the SCFHS.

Data collection tool

A total of 101 board residents and emergency physicians registered with the SCFHS participated in this study. An online survey was created and verified to meet the research purpose. The four components of the questionnaire were designed to collect socio-demographic data, information on how HINTS examination in diagnosing central causes of dizziness, the ability to conduct the test, and details of the physician's practice and disposition.

The reliability and validity analysis was done before administering the questionnaire by conducting a pilot test to assess its applicability. For each important research variable, the level of agreement was measured using a Likert scale ranging from 0 (strongly disagree) to 4 (strongly agree).

Study outcomes

The primary outcome of this study was the evaluation of the HINTS examination's knowledge, attitude, and practice of emergency physicians in Saudi Arabia. The secondary outcomes were identifying deficiencies in knowledge and determining factors contributing to the lack of knowledge, attitude, and practice of the HINTS examination in the ER.

Statistical analysis

The data was entered using Microsoft Excel software (Microsoft Corporation, Redmond, WA, USA). At the same time, the analysis was performed with SPSS Statistics version 27.0 (IBM Corp. Released 2020. IBM SPSS Statistics for Windows, Version 27.0. Armonk, NY: IBM Corp). The numbers and percentages were employed to represent the categorical variables, and the Monte Carlo test, the Pearson Chi-square (χ²), or the Fisher exact test were utilized to compare them. The continuous variables were presented as the mean ± standard deviation (SD). The threshold for statistical significance was set at p<0.05.

## Results

A total of 101 Saudi Arabian residents and emergency physicians registered with the SCFHS participated in this study. Table 1 shows that a high proportion of participants (68/101, 67.3%) had a good level of knowledge, a positive attitude (59/101, 58.4%), and good practice (82/101, 81.2%).

**Table 1:** Levels of knowledge, attitude, and practice of HINTS examination among emergency physicians (N=101) N: number, SD: standard deviation, HINTS: head impulse, nystagmus, and test of skew, Min: minimum, Max: maximum

	Knowledge		Attitude		Practice	
	Poor	Good	Negative	Positive	Poor	Good
N (%)	33 (32.7%)	68 (67.3%)	42 (41.6%)	59 (58.4%)	19 (18.8%)	82 (81.2%)

In this study, 44 (43.6%) of the emergency physicians in Saudi Arabia showed strong familiarity with the HINTS examination. However, many expressed uncertainty about its sensitivity compared to MRI for diagnosing AVS and its suitability for all vertigo patients. Most participants observed vertigo cases needing the HINTS examination, but many reported that ED colleagues seldom performed the test, and only seven (6.9%) felt confident conducting it. While over half felt undertrained, 80 (79.2%) advocated for teaching the HINTS examination to ED trainees. ED and consulting services were perceived as underutilizing the exam, highlighting a need for further education (Table 2).

**Table 2:** Knowledge, attitude, and practice of HINTS examination among emergency physicians (N=101) N: number, SD: standard deviation, HINTS: head impulse, nystagmus, and test of skew, ED: emergency department, AVS: acute vestibular syndrome, NA: no answers

	Strongly agree, N (%)	Agree, N (%)	Neutral, N (%)	Disagree, N (%)	Strongly disagree, N (%)	
						Familiar and knowledgeable about HINTS examination	44 (43.6%)	38 (37.6%)	13 (12.9%)	4 (4%)	2 (2%)	
HINTS examination is more sensitive than MRI in diagnosing AVS	19 (18.8%)	27 (26.7%)	31 (30.7%)	22 (21.8%)	2 (2%)	
HINTS examination can be applied to all patients complaining of vertigo	16 (15.8%)	35 (34.7%)	15 (14.9%)	24 (23.8%)	11 (10.9%)	
HINTS examination can be used to differentiate between peripheral and central vertigo	2 (2%)	1 (1%)	6 (5.9%)	35 (34.7%)	57 (56.4%)	
I see patients with vertigo requiring HINTS examination in my regular practice	NA	92 (91.1%)	NA	9 (8.9%)	NA	
Usually witness my ED colleagues performing HINTS examination in their practice	NA	40 (39.6%)	NA	61 (60.4%)	NA	
Instruction of ED trainee to perform HINTS examination	NA	80 (79.2%)	NA	21 (20.8%)	NA	
By discussing a case of acute-vestibular syndrome, they usually ask whether HINTS examination was performed or not	9 (8.9%)	19 (18.8%)	26 (25.7%)	36 (35.6%)	11 (10.9%)	
Consultation service on call is performing HINTS examination when evaluating AVS when attending ED patients	6 (5.9%)	19 (18.8%)	31 (30.7%)	36 (35.6%)	9 (8.9%)	
Confident when performing HINTS examination on my ED patient when applicable	7 (6.9%)	37 (36.6%)	26 (25.7%)	13 (12.9%)	18 (17.8%)	
HINTS assessment is mandatory when evaluating ED patients with AVS by ED physicians	22 (21.8%)	51 (50.5%)	21 (20.8%)	4 (4%)	3 (3%)	
HINTS examination is underutilized by most ED physicians	24 (23.8%)	56 (55.4%)	17 (16.8%)	1 (1%)	3 (3%)	
HINTS examination is underutilized by most of the consultation service on call	15 (14.9%)	47 (46.5%)	31 (30.7%)	7 (6.9%)	1 (1%)	
ED clinicians need to improve their knowledge and skills related to HINTS examination with more educational activities	26 (25.7%)	40 (39.6%)	8 (7.9%)	6 (5.9%)	21 (20.8%)	

Table 3 indicates that while the physician's position and certificate may be significant factors (p=0.017 and 0.028, respectively), age, gender, hospital sectors, and experience were not factors that contributed to the level of knowledge (p=0.349, 0.200, 0.535, and 0.120, respectively). Most ED residents in training (24/33, 72.7%) and most of the Saudi board of EM (19/33, 57.6%) demonstrated inadequate expertise in the HINTS examination.

**Table 3:** Some contributing factors of knowledge of HINTS examination among emergency physicians (N=101) * statistically significant at p<0.05 N: number, SD: standard deviation, HINTS: head impulse, nystagmus, and test of skew, ED: emergency department, EM: emergency medicine, GP: general practitioner, 1: Chi-square test, 2: Montecarlo test

Studied variables			Grades of knowledge		p-value	Test value
			Poor	Good		
Age in years	20-30	N (%)	24 (75.0%)	41 (60.3%)	0.349^1^	2.10
	31-40	N (%)	5 (15.6%)	18 (26.5%)		
	41-50	N (%)	3 (9.4%)	9 (13.2%)		
Gender	Female	N (%)	15 (45.5%)	22 (32.4%)	0.200^1^	1.64
	Male	N (%)	18 (54.5%)	46 (67.6%)		
Hospital sector	Public	N (%)	26 (78.8%)	57 (83.8%)	0.535^1^	0.38
	Private	N (%)	7 (21.2%)	11 (16.2%)		
Years of EM experience	0-5	N (%)	26 (78.8%)	46 (67.6%)	0.120^1^	4.23
	6-10	N (%)	1 (3.0%)	12 (17.6%)		
	>11	N (%)	6 (18.2%)	10 (14.7%)		
Position	ED resident in training	N (%)	24 (72.7%)	34 (50.0%)	0.017*^2^	10.45
	GP working full time in ED	N (%)	3 (9.1%)	1 (1.5%)		
	Senior registrar	N (%)	3 (9.1%)	22 (32.4%)		
	Registrar	N (%)	0 (0.0%)	3 (4.4%)		
	Consultant	N (%)	3 (9.1%)	8 (11.8%)		
Certificate	No board	N (%)	9 (27.3%)	5 (7.4%)	0.028*^2^	9.12
	Saudi Board of EM	N (%)	19 (57.6%)	42 (61.8%)		
	Non-Saudi Board of EM	N (%)	4 (12.1%)	13 (19.1%)		
	Both Saudi and non-Saudi boards of EM	N (%)	1 (3.0%)	8 (11.8%)		

Table 4 indicates that the level of attitude was not influenced by age, gender, hospital sector, experience, or position (p=0.127, 0.499, 0.190, 0.078, and 0.051, respectively). A notable proportion of physicians who hold the Saudi Board of EM certification (40/59, 67.8%) expressed a favorable view of the HINTS examination. Among examination physicians, certification may influence their attitude toward HINTS examinations (p=0.004).

**Table 4:** Some contributing factors of attitude of HINTS examination among emergency physicians (N=101) * Statistically significant at p<0.05 N: number, SD: standard deviation, HINTS: head impulse, nystagmus, and test of skew, ED: emergency department, EM: emergency medicine, GP: general practitioner, 1: Chi-square test, 2: Montecarlo test

Studied variables			Levels of attitude		p-value	Test value
			Negative	Positive		
Age in years	20-30	N (%)	32 (76.2%)	33 (56.9%)	0.127^1^	4.12
	31-40	N (%)	6 (14.3%)	17 (29.3%)		
	41-50	N (%)	4 (9.5%)	8 (13.8%)		
Gender	Female	N (%)	17 (40.5%)	20 (33.9%)	0.499^1^	0.46
	Male	N (%)	25 (59.5%)	39 (66.1%)		
Hospital sector	Public	N (%)	37 (88.1%)	46 (78.0%)	0.190^1^	1.72
	Private	N (%)	5 (11.9%)	13 (22.0%)		
Years of EM experience	0-5	N (%)	35 (83.3%)	37 (62.7%)	0.078^1^	5.10
	6-10	N (%)	3 (7.1%)	10 (16.9%)		
	>11	N (%)	4 (9.5%)	12 (20.3%)		
Position	ED resident in training	N (%)	27 (64.3%)	31 (52.5%)	0.051^2^	9.45
	GP working full time in ED	N (%)	4 (9.5%)	0 (0.0%)		
	Senior registrar	N (%)	7 (16.7%)	18 (30.5%)		
	Registrar	N (%)	1 (2.4%)	2 (3.4%)		
	Consultant	N (%)	3 (7.1%)	8 (13.6%)		
Certificate	No board	N (%)	12 (28.6%)	2 (3.4%)	0.004*^2^	13.20
	Saudi Board of EM	N (%)	21 (50.0%)	40 (67.8%)		
	Non-Saudi Board of EM	N (%)	5 (11.9%)	12 (20.3%)		
	Both Saudi and non-Saudi boards of EM	N (%)	4 (9.5%)	5 (8.5%)		

Table 5 indicates that the level of practice was not influenced by age, gender, hospital sector, experience, position, or credentials (p= 0.123, 0.281, 0.182, 0.380, 0.108, and 0.649, respectively).

**Table 5:** Some contributing factors of practice of HINTS examination among emergency physicians (N=101) N: number, SD: standard deviation, HINTS: head impulse, nystagmus, and test of skew, ED: emergency department, EM: emergency medicine, GP: general practitioner, 1: Montecarlo test, 2: Chi-square test, 3: Fisher exact test

Studied variables			Grades of practice		p-value	Test value
			Poor	Good		
Age in years	20-30	N (%)	16 (84.2%)	49 (60.5%)	0.123^1^	4.32
	31-40	N (%)	1 (5.3%)	22 (27.2%)		
	41-50	N (%)	2 (10.5%)	10 (12.3%)		
Gender	Female	N (%)	9 (47.4%)	28 (34.1%)	0.281^2^	1.16
	Male	N (%)	10 (52.6%)	54 (65.9%)		
Hospital sector	Public	N (%)	18 (94.7%)	65 (79.3%)	0.182^3^	1.78
	Private	N (%)	1 (5.3%)	17 (20.7%)		
Years of EM experience	0-5	N (%)	16 (84.2%)	56 (68.3%)	0.380^1^	1.95
	6-10	N (%)	1 (5.3%)	12 (14.6%)		
	>11	N (%)	2 (10.5%)	14 (17.1%)		
Position	ED resident in training	N (%)	15 (78.9%)	43 (52.4%)	0.108^1^	6.12
	GP working full time in ED	N (%)	1 (5.3%)	3 (3.7%)		
	Senior registrar	N (%)	1 (5.3%)	24 (29.3%)		
	Registrar	N (%)	1 (5.3%)	2 (2.4%)		
	Consultant	N (%)	1 (5.3%)	10 (12.2%)		
Certificate	No board	N (%)	4 (21.1%)	10 (12.2%)	0.649^2^	0.86
	Saudi Board of EM	N (%)	12 (63.2%)	49 (59.8%)		
	Non-Saudi Board of EM	N (%)	2 (10.5%)	15 (18.3%)		
	Both Saudi and non-Saudi boards of EM	N (%)	1 (5.3%)	8 (9.8%)		

## Discussion

Studies on the diagnostic efficacy of emergency physicians using the HINTS examination are ongoing. However, no studies have evaluated emergency physicians' knowledge and use of the HINTS examination in Saudi Arabia [9]. Therefore, this study aimed to assess the HINTS examination's knowledge, attitude, and practice among SCFHS-registered emergency physicians and board residents in Saudi Arabia.

In Canada, Byworth et al. [7] found that emergency physicians required education on how to apply the HINTS examination, with particular emphasis on its quality improvement initiatives. Nevertheless, the use of this approach in ED in the United Kingdom remains relatively low [10]. Quimby et al. [2] suggested that this suboptimal utilization can be attributed to either a lack of awareness of the test or physicians’ confidence in conducting and interpreting the HINTS examination. Zwergal et al. [11] discovered that only a small percentage of physicians were aware of the HINTS examination in Switzerland.

Many respondents were neutral regarding the sensitivity of the HINTS examination versus MRI in diagnosing AVS. According to Kattah et al. [9], the HINTS examination is more sensitive than an MRI for detecting posterior circulation stroke within the first 24 hours. Byworth et al. [7] reported that emergency physicians commonly order CT/CTA to evaluate vertigo because they cannot access MRI. While CT is useful for evaluating vertebrobasilar thrombi, dissection, and hemorrhage, it is less effective for detecting posterior circulation strokes, especially in the acute phase. Even when performed within 24 hours of symptom onset, MRI fails to diagnose up to 31% of posterior circulation strokes [9]. An acute brain MRI typically costs over $1,000 and requires at least 5-10 minutes of scan time, in addition to a waiting period of several hours to days. The HINTS examination may offer a rapid, cost-effective alternative to existing treatment in a context where efficiency and cost containment are paramount [2].

Previous research on using the HINTS examination by emergency physicians has shown a discrepancy in the level of support for two main schools of thought. Some studies have suggested that emergency physicians cannot accurately perform the HINTS examination [1,9,12]. However, other studies have not come to a negative conclusion about emergency physicians but instead have advocated for further research into the necessary education and training [5,13].

In a systematic review, Ohle et al. [1] reported that emergency physicians using standard equipment can complete the HINTS examination with a sensitivity of 83% and a specificity of 44%. However, this review included vascular neurologists, otologists, and ophthalmologists with fellowship training as examiners [9,12]. Nevertheless, studies incorporating elements of the HINTS examination, such as the head-impulse test [14] or head-impulse test and nystagmus assessment [15], found emergency medicine physicians with high sensitivity (93-100%) and specificity (87-96%) for identifying central causes. These findings suggest that emergency physicians can effectively perform and interpret the HINTS examination. Gerlier et al. [16] was the only study conducted among experienced emergency physicians that reported high sensitivity (97%) and lower specificity (65%).

More than half of our participants strongly disagreed with using the HINTS examination to differentiate between central and peripheral vertigo. Zwergal et al. [11] found that a small percentage of physicians recognized the HINTS examination (+) as a crucial bedside test for accurately distinguishing between peripheral and central causes of acute persistent vertigo and dizziness. In retrospective cohort research, the HINTS examination has little diagnostic utility in ED settings, and it is often administered to individuals who do not match the eligibility requirements. The study also discovered that the HINTS examination could not identify any cases of dizziness with a central etiology [5]. Moreover, studies that depend on conventional methods for eliciting vertigo found that 44% of patients who present with dizziness are misdiagnosed, and 35% of stroke patients who report dizziness are misdiagnosed in the ER [17-19].

Gerlier et al. [16] found that their ER respondents expressed concern about the high percentage of false-positive exams. Indeed, false-positive results from a "central HINTS" examination are more frequent than false-negative results from a "peripheral HINTS" examination. Byworth et al. [7] thought that catch-up saccades are extremely challenging to detect, particularly in the absence of video-nystagmography, which leads to an excessive number of erroneous "central HINTS" examination results. Kerber et al. [12,17] proposed that a test should be designed to prioritize sensitivity over specificity. This is because the consequences of a false negative result are more severe than those of a false positive result.

Dizziness is a challenging condition to diagnose, and only 36.6% of respondents indicated that they felt confident using the HINTS examination to correctly detect stroke in patients experiencing acute vertigo. According to Warner et al. [3], there was a lack of high trust among clinicians in executing the HINTS examination. The restrictions on physical contact and group training during the COVID-19 pandemic have led to a lack of training and a surge in demand for online learning environments, despite reservations about the quality of accessible materials.

In the current study, the HINTS examination was underutilized by most emergency physicians and consultants. Additionally, they required further education to enhance their knowledge and skills. Several factors were identified, including a lack of awareness of when to use the examination, the components of the examination itself, how to complete and interpret the examination components, and its result. Older physicians perceived the head-impulse test as challenging, painful, and potentially dangerous [7]. The HINTS examination has been validated since 2009. However, because new medical data is slowly being translated into knowledge, older physicians may not be as familiar with or comfortable performing the HINTS examination [6]. Furthermore, the head-impulse test, the initial phase of the three-component HINTS examination, has been identified as a potential concern regarding its practicality and safety for some emergency physicians [7]. The procedure can be performed with minimal discomfort by thrusting the head towards the midline rather than away and avoiding head rotations greater than 15-20% [9,20-22]. Therefore, training is required on appropriately administering the HINTS examination, particularly the head-impulse test [7]. It is thought that there are insufficient opportunities in EM settings to develop the necessary skills for evaluation [16]. Newer videos of head-impulse tests and using them in practice are necessary [2,5,23]. Further research is recommended into the education and training needed for emergency physicians to conduct the HINTS examination [6].

In the current study, male physicians in their younger years, emergency physicians with experience of less than five years, public hospital physicians, ED residents in training, and those who have a certificate of the Saudi Board of EM were more likely to possess strong knowledge, a positive attitude, and a high level of practice regarding the HINTS examination. Physician positions and certifications were only statistically associated with the knowledge of the HINTS examination. However, age, gender, hospital sector, and years of experience were not statistically associated with knowledge, attitude, and practice. This could be attributed to the relatively small sample size. Byworth et al. [7] reported that most respondents were in the younger age range of 25-34. This may be related to the recent introduction of the specialty in Saudi Arabia and the rest of the world. It is also not surprising to find newly qualified physicians eager to take advantage of opportunities to participate in professional development activities. The overrepresentation of male emergency physicians in the survey may reflect their dominance in the Saudi Arabian EM field. Approximately 3,000 primary care centers are dispersed throughout Saudi Arabia, collectively accounting for 80% of the nation's healthcare provision. A publicly funded health system largely facilitates this. The private sector is responsible for 20% of healthcare delivery [24].

Compared to other Middle Eastern nations, the EM sector in Saudi Arabia is more developed and regarded as a reputable specialty in tertiary care facilities [25]. It was also the first nation in the region to establish a certification board, the Saudi Board of EM, which was established in 2001 based on American standards. The Saudi Society of EM was founded in 2007 to promote and legitimize the specialty. Moreover, the Saudi Board has demonstrated excellence in assisting Middle Eastern Arab boards of EM in their development [26].

The SCFHS defines an EM consultant as a board-certified emergency physician who has completed EM residency training. Residency training was obtained locally or abroad in other nations, including the USA, Canada, Australia, South Africa, and France, by practicing EM attending physicians. The Arab Board of EM, which the Arab Board of Health Specialization governs, certifies a different group of emergency physicians. Nevertheless, Saudi Arabia is experiencing a severe shortage of licensed emergency physicians. There are still certain hospitals and even cities where the EDs lack a qualified emergency physician. Instead, general practitioners or physicians with internal medicine or general surgery training lead the ED teams [8].

This survey data will enable the development of evidence-based targeted education campaigns in the future. Prospective investigations may comprise experiments intended to tackle issues related to the reliability and applicability of the test. These studies could help boost the use of the HINTS examination by having skilled EM doctors provide it to suitably chosen patients without needing assistive technology. A more accurate and comprehensive utilization of the HINTS examination would enhance the diagnostic precision of stroke identification, facilitate the management of its subsequent effects, and prevent subsequent strokes. Furthermore, the absence of imaging requirements would expedite the discharge of patients with vestibular neuritis.

This study has several limitations. In Saudi Arabia, there was no accessible registry to obtain the information of all emergency physicians and specialists. This highlights the difficulty in determining the response rate and the exact desired total sample size. Furthermore, as an inherent limitation of the research design, participants' views were influenced by numerous variables related to their previous experience with simulation and current intentions. Thus, future large-scale multicenter research is needed.

## Conclusions

In Saudi Arabia, male physicians in their younger years, those with less than five years of EM experience, public hospital physicians, ED residents in training, and those holding the Saudi Board of EM certificate were more likely to have strong knowledge, positive attitudes, and high levels of practice regarding the HINTS examination. However, there are several knowledge gaps regarding its sensitivity, how and when to use it, and lack of confidence and underutilization of the HINTS examination. Therefore, several physicians require additional educational efforts. Further research is needed to determine the most effective method to educate and train physicians on the HINTS examination.
